# ROCK inhibition modulates the senescence‐associated secretory phenotype (SASP) in oral keratinocytes

**DOI:** 10.1002/2211-5463.13012

**Published:** 2020-11-06

**Authors:** Sven Niklander, Deepti Bandaru, Daniel W. Lambert, Keith D. Hunter

**Affiliations:** ^1^ Unit of Oral and Maxillofacial Medicine, Pathology and Surgery University of Sheffield Sheffield UK; ^2^ Departamento de Cirugia y Patologia Oral Facultad de Odontologia Universidad Andres Bello Viña del Mar Chile

**Keywords:** Rho kinase, ROCK inhibitors, SASP, senescence, Y‐27632

## Abstract

Senescent cells accumulate in different organs and develop a senescence‐associated secretory phenotype (SASP), associated with the development of age‐related pathologies. The constitution of the SASP varies among cell types and with the method of senescence induction; nevertheless, there is substantial overlap among SASPs, especially the presence of pro‐inflammatory cytokines such as IL‐1β, IL‐1α, IL‐6 and IL‐8. These cytokines are highly conserved among SASPs and are implicated in the development of several cancers. Here, we report that ROCK inhibition by Y‐27632 reduces levels of IL‐1α, IL‐1β, IL‐6 and IL‐8 secreted by senescent normal and dysplastic oral keratinocytes without affecting the permanent cell growth arrest. The data indicate some inflammatory genes downregulated by Y‐27632 remain downregulated even after repeated passage in the absence of Y‐27632. We propose ROCK kinase inhibition as a novel alternative to current strategies to modulate the inflammatory components of the SASP, without compromising the permanent cell growth arrest. This observation potentially has wide clinical applications, given the involvement of senescence in cancer and a wide range of age‐related disease. It also suggests care should be exercised when using Y‐27632 to facilitate cell expansion of primary cells, as its effects on gene expression are not entirely reversible.

## Graphical abstract

Senescent cells accumulate in different organs and develop a senescence‐associated secretory phenotype (SASP), associated with the development of age‐related pathologies, including cancer. Here, we report that ROCK inhibition by Y‐27632 reduces levels of important SASP factors (IL‐1α, IL‐1β, IL‐6 and IL‐8) secreted by senescent normal and dysplastic oral keratinocytes without affecting the permanent cell growth arrest.

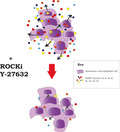

**Abbreviations**
IL: interleukin
IL‐1R1: interleukin 1 receptor 1
NF‐κB: nuclear factor kappa‐light‐chain‐enhancer of activated B cells
NOK: normal oral keratinocyte
OD: oral dysplasia
p38 MPAK: p38 mitogen‐activated protein kinase
ROCK: Rho kinase
SA‐β‐Gal: senescence‐associated beta‐galactosidase
SASP: senescence‐associated secretory phenotype

Senescence is a cellular state characterized by permanent cell growth arrest in response to different stressors [1]. It is considered a potent tumour‐suppressor mechanism [2, 3, 4], because it can stop cells with somatic mutations (considered as precancerous cells) from dividing and acquiring further mutations that could enable replicative immortality and cancer development [5]. Despite this, senescence is associated with the development of age‐related diseases, including cancer [6, 7]. Senescent cells have a characteristic gene expression profile [8] and secrete more than 40 pro‐inflammatory molecules (cytokines, chemokines, growth factors and proteases) involved in intercellular signalling, a phenotype recognized as the senescence‐associated secretory phenotype (SASP) [7, 9]. The transcriptome and secretome of the SASP vary among cell types and with aetiology of senescence (telomere shortening, oncogene activation, etc.) [10, 11], which reflects the heterogeneity of the SASP. Nevertheless, there is substantial overlap among SASPs, especially with pro‐inflammatory cytokines such as interleukin (IL)‐1β, IL‐1α, IL‐6 and IL‐8, which are highly conserved among SASPs [12].

In the context of tumorigenesis, senescence can be a beneficial response, preventing cells with oncogenic mutations dividing, thus preventing cancer. But the development and dysregulation of the SASP can have the opposite effects. Thus, different approaches, using a variety of agents to target the SASP or key SASP factors have been employed, which includes the use of metformin [13], C75 (a fatty acid synthase inhibitor) [14], p38MAPK inhibitors [15], rapamycin (mammalian target of rapamycin inhibitor) [16] and anakinra (exogenous interleukin 1 receptor antagonist) [17].

Rho kinase (ROCK) is a downstream target of the small GTP‐binding protein Rho and has two isoforms, ROCK1 and ROCK2 [18, 19]. ROCKs belong to the serine threonine kinase family which modulate various important cellular functions, such as cell shape, motility, secretion, cellular growth, apoptosis, gene expression and cell cycle progression [20]. Because of that, ROCK inhibitors have been proposed as novel therapeutics in a number of clinical scenarios, including asthma [21], glaucoma [22], systemic lupus erythematosus [23], cardiovascular diseases [24, 25], bone healing [26] and cancer [27].

IL‐1 signalling is essential for the development of the SASP and its oncogenic properties [28]. Both IL‐1α and IL‐1β are able to increase secretion of SASP factors such as IL‐6 and IL‐8, which have known oncogenic properties [7, 28, 29], and IL‐1α inactivation in senescent cells impairs tumour progression [28]. Recently, the ROCK inhibitor Y‐27632 was shown to reduce IL‐1‐induced IL‐8 secretion in Caco‐2 cells, by reducing the phosphorylation of p38 MAPK [30]. Similar to this, another study reported Y‐27632 reduced LPS‐induced IL‐6 and IL‐8 secretion in human gingival fibroblasts, by inactivating the nuclear factor kappa‐light‐chain‐enhancer of activated B cells (NF‐κB) and p38 MAPK pathways. Due to its ability to inhibit IL‐1 signalling and the importance of the IL‐1 pathway for the development of the SASP, we investigated the effects of Y‐27632 on presenescent and senescent cells and examined whether Y‐27632 could modulate the expression of commonly expressed SASP factors related to cancer development. Y‐27632 is commonly used in cell biology to facilitate the manipulation of primary cell cultures, as treated cells are able to proliferate indefinitely without genetic alterations [31]. Nevertheless, it is not known how reversible its effects are and what other consequences may be in the cells. Thus, we also explored the reversibility of Y‐27632 effects.

## Results

### Y‐27632 modifies the SASP from senescent normal and dysplastic oral keratinocytes

**Fig. 1:**
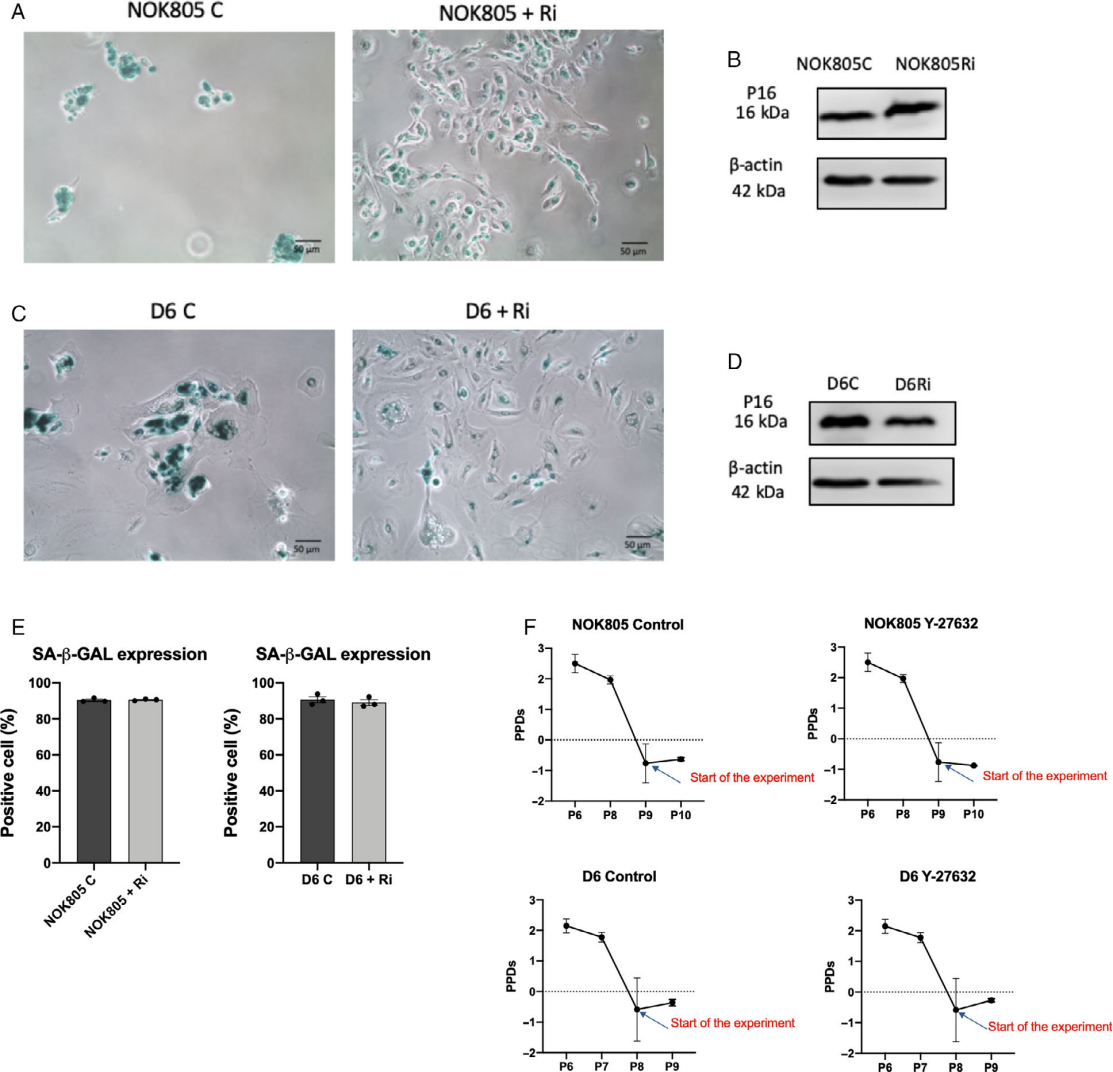
Y‐27632 does not rescue oral keratinocytes from senescence. (A, C) SA‐β‐Gal activity decreases after Y‐27632 treatment in both NOK805 (A) and D6 (C) senescent cells, but cells did not recapitulate cell growth. Scale bars are 50 μm. (B, D) p16 expression in senescent NOK805 (B) and D6 cells (D) does not change after Y‐27632. (E) SA‐β‐Gal quantification in senescent NOK805 and D6‐treated and nontreated cells. Data are shown as mean ± SEM. (F) Population doublings of senescent NOK805 and D6‐treated and nontreated cells. Data are shown as mean ± SEM. *N* = 3 independent experiments.

To analyse the effects of ROCK inhibition on senescent cells, we treated normal (NOK805) and dysplastic (D6) senescent oral keratinocytes with a commonly used ROCK inhibitor, Y‐27632 [32]. First, we wanted to assess whether Y‐27632 was able to rescue senescent keratinocytes from permanent cell growth arrest (Fig. 1A‐D). In both senescent NOK805 and D6 cell cultures, a decrease in senescence‐associated beta‐galactosidase (SA‐β‐Gal) staining was noted, but no differences in SA‐β‐Gal‐positive cells (*P *> 0.05) nor consistent changes in p16 expression between Y‐27632‐treated and nontreated cells were observed (Fig. 1E). In both Y‐27632‐treated and nontreated cells (for both NOK805 and D6 cells), there were no increases in population doublings after exposure to Y‐27632 or nothing for 6 days, confirming that in neither cell type Y‐27632 was able recapitulate cell proliferation (Fig. 1F).

**Fig. 2:**
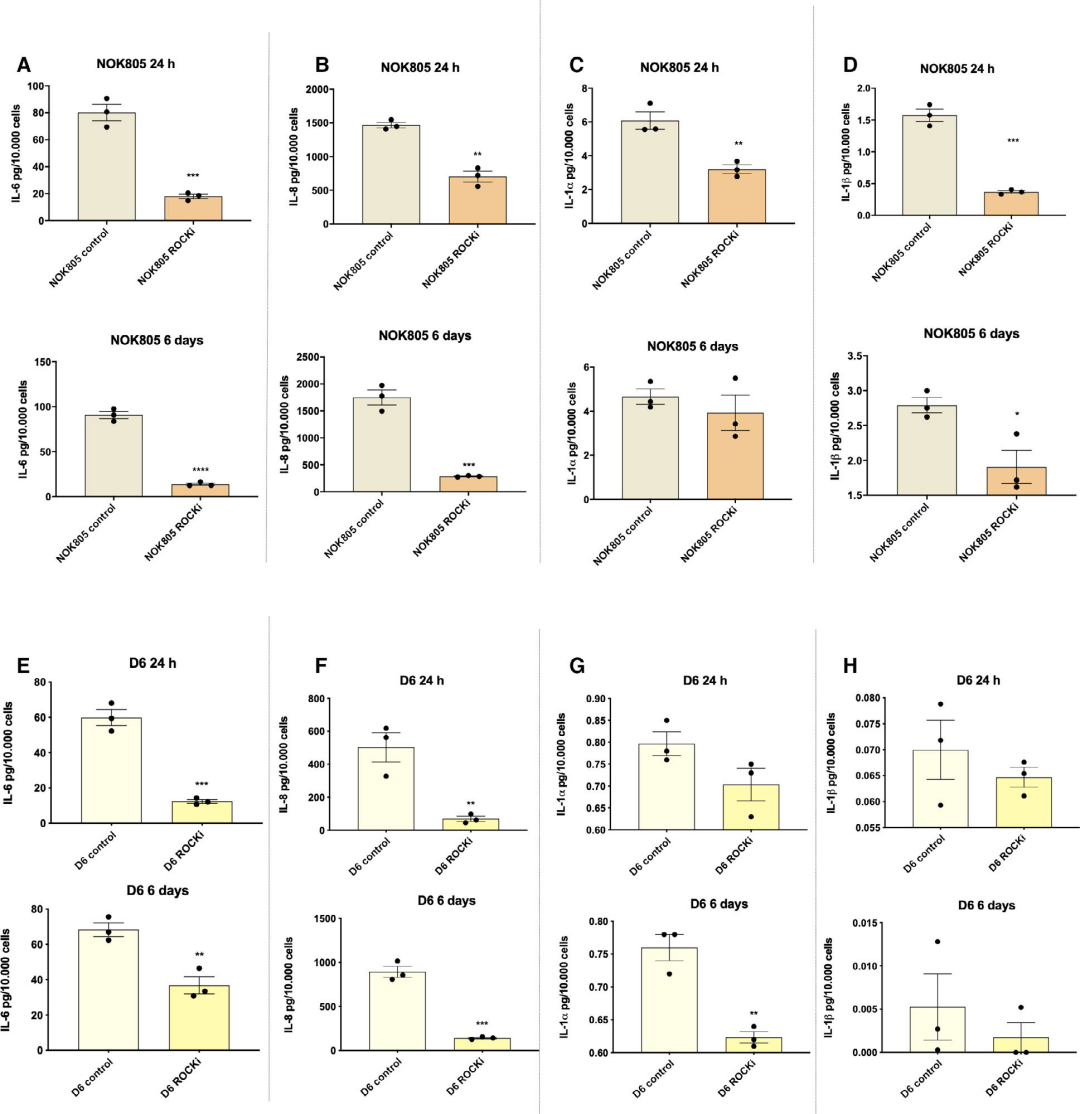
Y‐27632 modifies the SASP from senescent normal and dysplastic oral keratinocytes. (A‐H) Effect of Y‐27632 on secreted levels of IL‐6, IL‐8, IL‐1α and IL‐1β from senescent NOK (A‐D) and senescent dysplastic oral keratinocytes (E‐H). Data are shown as mean ± SEM (*N* = 3 independent experiments, *n* = 2 technical replicates). Two‐tailed *t*‐test was used to calculate the exact *P* value. Data information: *P < 0.05, **P < 0.005 and ***P < 0.0005.

To study the effects of Y‐27632 on the SASP, senescent NOK805 and D6 cells were treated for a period of 24 h or 6 days with Y‐27632 (10 μg·mL^−1^). Conditioned media (CM) were collected, and secreted levels of commonly expressed SASP factors (IL‐6, IL‐8, IL‐1α and IL‐1β) [12] were analysed using ELISA. In senescent normal oral keratinocytes (NOK), Y‐27632 reduced IL‐6, IL‐8 and IL‐1β levels significantly at both time intervals (*P* < 0.05), apart from IL‐1α levels which were decreased only after 24 h of treatment (Fig. 2A‐D). In senescent dysplastic oral keratinocytes, there was a significant decrease in IL‐6 and IL‐8 levels after 24 h and 6 days of treatment with Y‐27632 (*P* < 0.05). IL‐1α levels also decreased but only after 6 days of treatment (*P* < 0.05), and no significant changes in IL‐1β levels were observed (Fig. 2E–H).

### Changes in gene expression induced by Y‐27632 are not always reversible

**Fig. 3:**
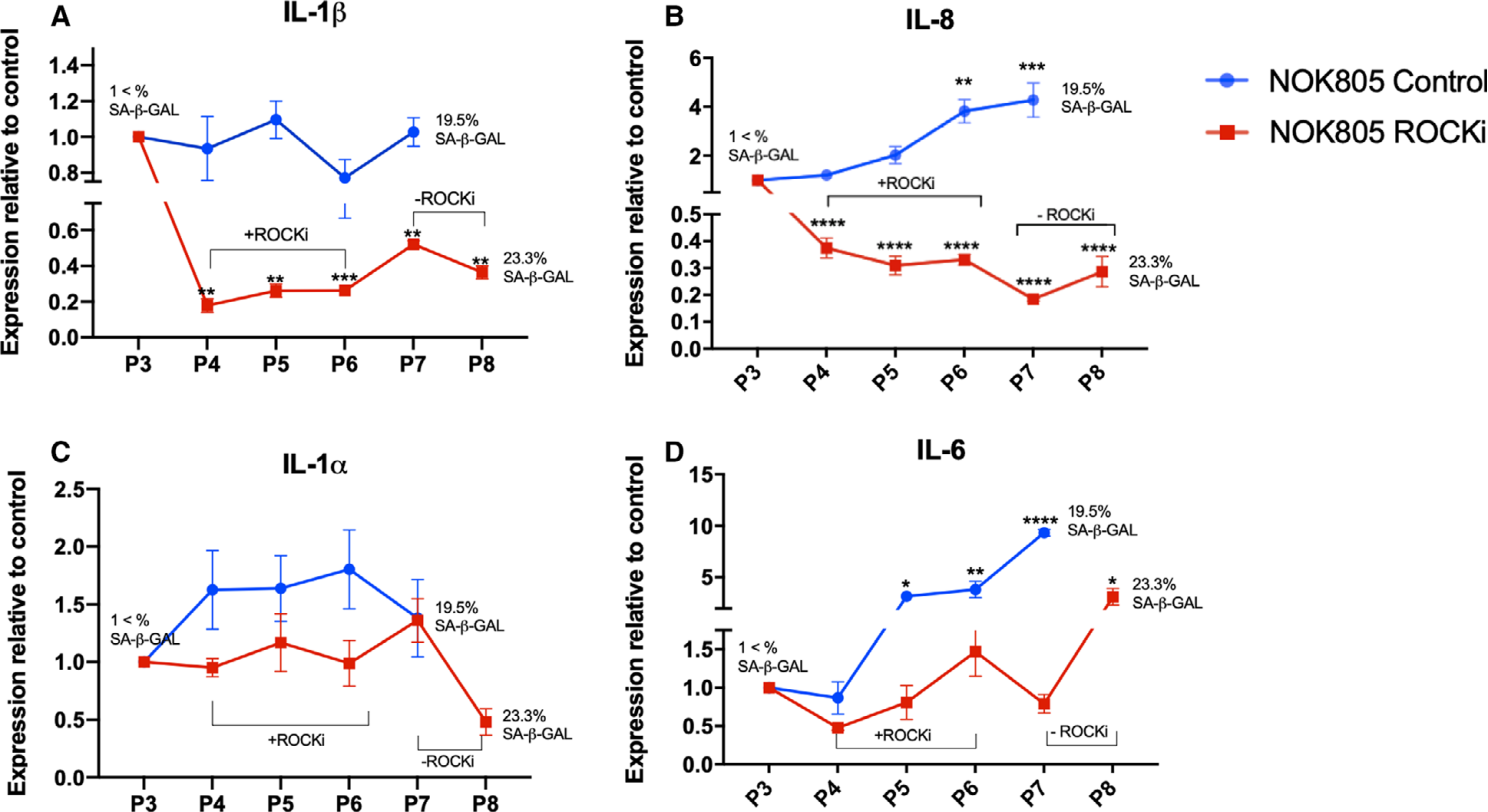
Y‐27632 modifies expression of IL‐1‐related genes which is not always reversible upon drug withdrawal. (A‐D) IL‐1β (A), IL‐8 (B), IL‐1α (C) and IL‐6 (D) gene expression during and after Y‐27632 treatment in NOKs. Data are shown as mean ± SEM (*N* = 3 independent experiments, *n* = 3 technical replicates). One‐way ANOVA with multiple comparisons was used to calculate the exact *P* value. Data information: *P < 0.05, **P < 0.005, ***P < 0.0005 and ****P < 0.0001.

Next, we assessed whether changes in SASP cytokine expression induced by Y‐27632 are reversible upon Y‐27632 removal from culture conditions. For this purpose, we used primary NOK (NOK805). NOK805 cells were passaged once without Y‐27632, three times with 10 μg·mL^−1^ of Y‐27632 and then two more times without Y‐27632. Control cells (NOK805 cells without exposure to Y‐27632) were passaged the same number of times. Primary keratinocytes have a limited lifespan in culture, usually senescing between 8 and 12 passages. Because of that, SA‐β‐Gal activity was quantified at start and endpoint. Y‐27632 significantly reduced IL‐1β and IL‐8 mRNA transcripts, which remained significantly lower even two passages after the drug was removed (Fig. 3A,B). No significant changes in IL‐1α and IL‐6 mRNA transcripts were observed during or after treatment with Y‐27632 (Fig. 3C,D).

### Chemically immortalized keratinocytes senesce after Y‐27632 removal from culture conditions

**Fig. 4:**
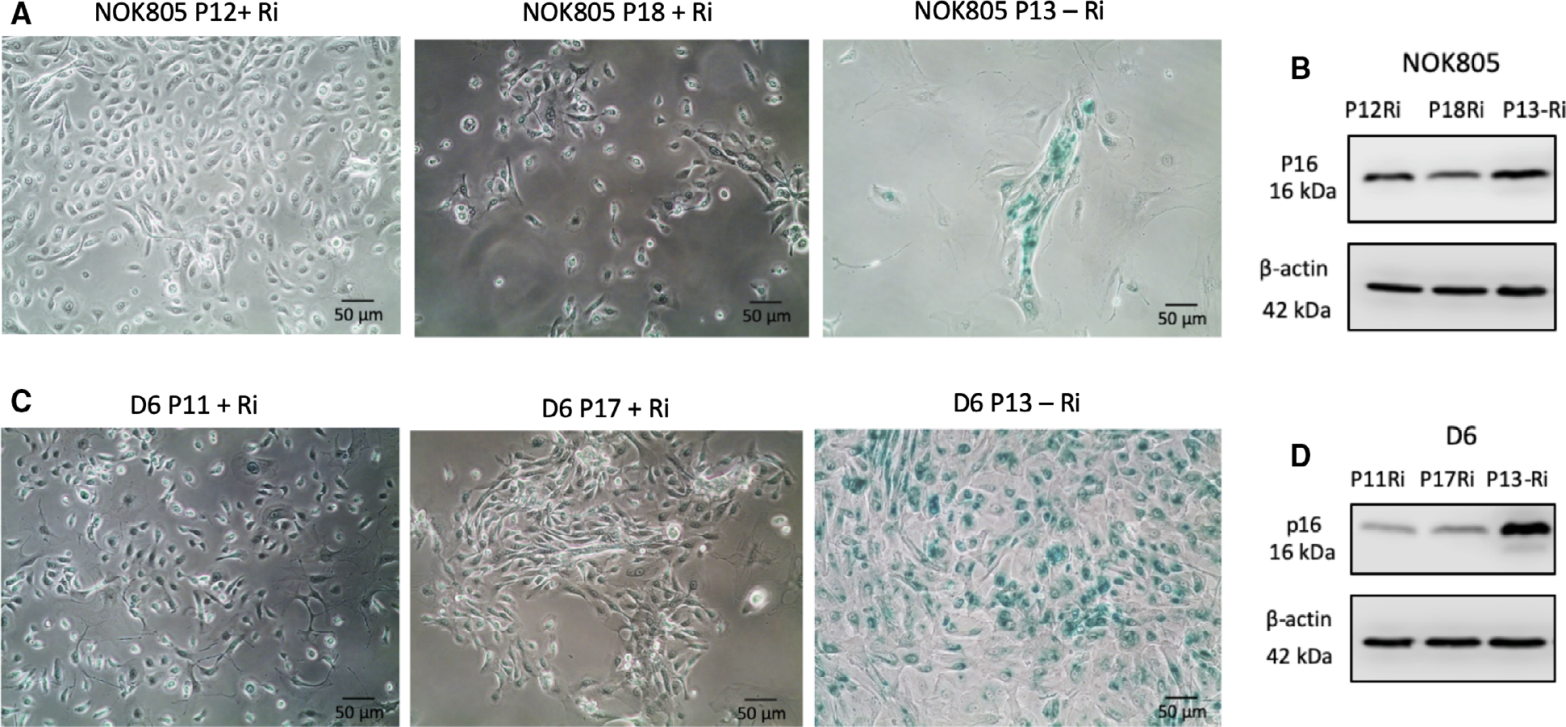
Oral keratinocytes immortalized with Y‐27632 senesce after Y‐27632 withdrawal. (A–D) Both NOK805 (A, B) and D6 (C, D) cells senesce after removal of Y‐27632 from culture conditions, as shown by the increase in SA‐β‐Gal (A, C) activity and p16 expression (B, D). In the presence of Y‐27632, NOK805 and D6 cells were still proliferative at passage 18 and 17, respectively (A, C). Scale bars are 50 μm (*N* = 3 independent experiments).

When studying the effects of Y‐27632 on gene expression, we made the observation that SA‐β‐Gal activity remained very low or undetectable when the cells were grown under Y‐27632 treatment. Once Y‐27632 was removed from culture conditions, a progressive increase in SA‐β‐Gal activity was observed, suggesting a reactivation of the senescence programme. Although one study reported cell growth arrest after Y‐27632 removal [33], it was not demonstrated that the growth arrest reported was senescence. Thus, we assessed the capabilities of cells grown in the presence of Y‐27632 to senesce after drug removal. Normally, NOK805 and D6 cells senesce at passages 10 and 8, respectively. We added 10 μg·mL^−1^ of Y‐27632 to the growth media of NOK805 and D6 at passages 2 and 3, respectively, and cultured them to passages 18 and 17 (cells were still proliferative at this point) with no evidence of SA‐β‐Gal activity (Fig. 4). We removed Y‐27632 from culture conditions at passages 12 (NOK805) and 11 (D6) and observed cessation of cell growth after 1 passage in NOK805 and 2 passages in D6. The cell growth arrest corresponded with high SA‐β‐Gal activity (> 90%) and p16 expression in both cell cultures (Fig. 4).

## Discussion

During ageing, senescent cells accumulate in different organs and develop a secretory phenotype, known as the SASP, which has been related to the development of age‐related pathologies [34]. During early stages, components of the SASP facilitate tissue repair, immune surveillance and remodelling [35], but in advanced stages, the persistence of the SASP negatively impacts the microenvironment by enabling a chronic inflammatory state dominated by IL‐1, IL‐6 and IL‐8 [28] through cell and non‐cell‐autonomous effects [36]. It has been experimentally demonstrated that some senescent cells, such as fibroblasts, can promote carcinogenesis of keratinocytes via factors present in the SASP [6, 37]. Coppe *et al*. [7] showed that the SASP from surrounding fibroblasts can induce epithelial‐to‐mesenchymal transition and invasion of premalignant breast epithelial cells by a paracrine mechanism dependent on IL‐6 and IL‐8. Similar results have been shown elsewhere [38]. The SASP also favours the emergence, maintenance and migration of cancer stem cells [39]. All of these reports provide evidence of the pro‐tumorigenic actions attributed to the SASP through IL‐6 and IL‐8. Thus, the modulation of SASP factors has promising clinical implications for the treatment of premalignancies and cancer.

Various attempts have been made to reduce the deleterious effects of senescent cells, whether by selective elimination of cells or by controlling the expression of specific SASP factors. Here, we report the ROCK inhibitor Y‐27632 decreases release of IL‐6 and IL‐8 SASP by senescent epithelial cells, which is likely to be a consequence of IL‐1 inhibition. Both IL‐1α and IL‐1β are crucial for the development of the SASP and are regulators of IL‐6 and IL‐8 secretion through NF‐κB activation [28, 40, 41]. IL‐1α regulates the SASP via interleukin 1 receptor 1 (IL‐1R1), as depleting cells of interleukin 1 receptor‐associated kinase 1 (a downstream kinase recruited after IL‐1R1 activation) reduce IL‐6 levels even after IL‐1 stimulation [42]. IL‐1α depletion also reduces NF‐kB activity, which is important for IL‐6 and IL‐8 secretion [42]. We observed Y‐27632 reduces IL‐1α levels in both normal and dysplastic cell cultures. A significant decrease in IL‐1β levels was also observed but only in NOK, as in dysplastic oral keratinocytes the decrease did not reach statistical significance. This is probably because IL‐1β levels were almost undetectable in senescent dysplastic cells (between 0.005 and 0.07 pg/10 000 cells). Importantly, we also showed that senescent cells treated with Y‐27632 do not restart proliferation, which is important if we want to modify the SASP without compromising the permanent cell growth arrest (desirable in the context of cancer, but also in other age‐associated pathologies) [43].

Y‐27632 is commonly used in cell culture as a facilitator of cell growth of primary keratinocytes due to its ability to enhance proliferation and to prolong cell lifespan. This is achieved by downregulation of keratinization and epithelial cell differentiation genes, and by upregulation of DNA replication, RNA processing, cell cycle and division genes [33]. It has been previously reported that Y‐27632 can affect IL‐1 signalling in proliferating cells [30, 44], but whether its effects are reversible upon drug withdrawal has not been assessed. This is of importance as when Y‐27632‐treated cells are used for experimentation, Y‐27632 is usually removed from cell culture conditions 24 h prior experimentation, with the assumption that the effects of the drug wear off after that time. We demonstrate that gene expression affected by Y‐27632 does not recover immediately after Y‐27632 withdrawal. In our study, IL‐1β and IL‐8 RNA transcript levels did not recover even after passaging the cells twice after Y‐27632 was removed (at least 10 days in culture). This has to be taken into consideration when using cells previously cultured with Y‐27632 for downstream experiments, as transcription of some genes might not recover to pretreatment levels, with likely phenotypic consequences.

The addition of Y‐27632 to the growth media in combination with a 3T3 fibroblast feeder layer has been shown to indefinitely prolong keratinocyte lifespan [31, 45], but the indefinite lifespan is conditional on the presence of both [31]. Nevertheless, it is unknown whether keratinocytes previously treated with Y‐27632 are able to senesce once the drug has been removed. There is only one report showing that after Y‐27632 removal keratinocytes stopped proliferating, but it is unclear whether the growth arrest was due to terminal differentiation, quiescence or senescence, as no senescence markers were assessed [33]. This is of importance as it would render Y‐27632 suitable for use in experiments aiming to elucidate mechanisms of senescence in primary cells. To date, there is no single universal marker that can identify senescent cells from nonproliferating, terminally differentiated or quiescent cells. Instead, a combination of multiple markers is used to identify senescent cells. We assessed senescence by evaluating SA‐β‐Gal activity and p16 expression, two commonly used markers to identify senescent cells [46]. We showed that keratinocytes previously treated with Y‐27632 enter a nonproliferative phase shortly after Y‐27632 has been removed from culture conditions, with high SA‐β‐Gal activity and high p16 expression, consistent with a senescence growth arrest.

To summarize, we showed that ROCK kinase inhibition reduces the abundance of IL‐1α, IL‐1β, IL‐6 and IL‐8 released by senescent cells, without compromising the permanent cell growth arrest. Our data therefore suggest that Y‐27632 is able to suppress the SASP, which could have wide clinical applications in cancer and other age‐associated diseases. Finally, our results demonstrate care has to be taken when using Y‐27632 to facilitate cell expansion of primary cells, as its effects on gene expression are not entirely reversible.

## Materials and methods

### Cell culture

Isolation of primary NOK (NOK805) was done under the University of Sheffield ethics (approval reference 3463). The experiments were undertaken with the understanding and written consent of each subject. The study methodologies conformed to the standards set by the Declaration of Helsinki.

The mortal dysplastic oral keratinocytes (D6) used in this study has been described previously [47]. Both D6 and NOK805 cells were grown using lethally irradiated (a) 3T3 feeders as described previously [48] in low‐glucose Dulbecco’s modified Eagle medium (Sigma‐Aldrich, Gillingham, UK) containing 10% vol/vol FBS (HyClone FetalClone II Serum; Fisher Scientific, Pittsburgh, NH, USA) and 21% vol/vol F12 nutrient mix (Sigma‐Aldrich), supplemented with 2 mm l‐glutamine (Sigma‐Aldrich), 0.25 μg·mL^−1^ adenine, 100 μg·mL^−1^ penicillin and 100 U·mL^−1^ streptomycin (Sigma‐Aldrich),10 ng·mL^−1^ epidermal growth factor (Sigma‐Aldrich), 1 mg·mL^−1^ human insulin (Sigma‐Aldrich), 4 μg·mL^−1^ hydrocortisone, 1.36 ng·mL^−1^ and 5 μg·mL^−1^ 3, 3, 5‐tri‐iodothyronine/apo‐transferrin and 0.7 mm Na pyruvate (Sigma‐Aldrich). All cell cultures were incubated at 37 °C with 5% CO_2_ and checked daily. Medium was replaced every 2–3 days, and cells were passaged when ~ 80% of confluency was achieved.

### Senescence‐associated β‐galactosidase staining

Twenty thousand cells per well were seeded in a 12‐well plate and left to adhere overnight. Senescence‐associated β‐galactosidase activity was assessed using a Senescence Detection Kit (ab65351; Abcam, Cambridge, UK) following the manufacturer’s recommendations.

### Treatment of proliferative oral keratinocytes with Y‐27632

Y‐27632 (Abcam) was added to the growth media at a concentration of 10 μg·mL^−1^ and replaced every 2–3 days as described previously [49].

### Treatment of senescent cells with Y‐27632

NOK805 and D6 cells were grown until replicative senescence was achieved (confirmed by cessation of cell growth, morphological changes and a SA‐β‐Gal staining > 95% for each cell type). After confirmation, both NOK805 and D6 cells were treated for a period of 24 h or 6 days with 10 μg·mL^−1^ of Y‐27632 (Abcam) and CM were collected.

### Enzyme‐linked immunosorbent assay (ELISA)

Secreted IL‐6, IL‐8, IL‐1β and IL‐1α were quantified using ELISA DuoSet from R&D Systems (Minneapolis, MN, USA) for human IL‐6, IL‐8, IL‐1β and IL‐1α detection. The procedure was performed following the manufacturer’s recommendation using 96‐well plates. Absorbance was read at 450 nm within 30 min of adding the stop solution. Wavelength correction was done subtracting absorbance at 570 nm from absorbance at 450 nm.

### Quantitative (q)PCR

Total RNA was extracted from cell pellets using the Isolate II RNA Mini Kit (Bioline, London, UK) RNA Extraction Kit following manufacturer’s instructions. RNA was quantified using a NanoDrop 1000 Spectrophotometer (Thermo Fisher Scientific, Cambridge, UK). Five hundred nanograms of isolated RNA was reverse‐transcribed using the High Capacity cDNA Reverse Transcription Kit (Applied Biosystems, Foster City, CA, USA) following manufacturer's protocol using a Peltier thermal cycler (MJ Research, San Diego, CA, USA). cDNA was then stored at −20 °C.

Gene expression was quantified with a Rotor‐Gene Q Real‐time PCR cycler (Qiagen, Manchester, UK) using TaqMan chemistry. Quantification was calculated using delta CT values normalized to B2M. Each reaction was performed in triplicate. All reactions were performed in total volumes of 10 μL loading 500 ng of cDNA. The standard thermal cycle settings for a reaction consisted in 40 cycles including a melt curve analysis (when using SYBR Green). One cycle consisted of 95 °C for 10 s, 60 °C for 15 s and 72 °C for 20 s. TaqMan probes were bought from Thermo Fisher Scientific and corresponded to IL‐1α (Hs: 00174092), IL‐1β (Hs: 01555410_m1), IL‐6 (Hs: 00985639), IL‐8 (Hs: 00174103) and B2M (Hs: 4325797).

### Western blotting

Protein was extracted by dissolving the cell pellets on an appropriate volume of lysis buffer on ice. Lysis buffer that consisted in one tablet of complete mini‐EDTA‐free protease inhibitor cocktail (Roche, Basel, Switzerland) and one tablet of phosphatase inhibitors (PhosSTOP; Roche) dissolved in 10 mL RIPA Buffer (Sigma‐Aldrich). Cell suspensions were left for 30 min on ice and centrifuged at 19 700 ***g*** for 10 min at 4 °C. The supernatants were stored at −20 °C, and the pellets were discarded. Protein quantification was done using the bicinchoninic acid assay (Thermo Fisher Scientific) according to the manufacturer’s protocol using a Tecan spectrophotometer (Spark, Männedorf, Switzerland).

Twenty micrograms of protein was mixed with 2× SDS lysis buffer, heated for 5 min at 95 °C and then loaded into 12% SDS/PAGE gels. Gels were run for ≈ 90 min at 150 V and transferred into nitrocellulose membranes using the Trans‐Blot® Turbo™ Transfer System (Bio‐Rad, Deeside, UK) according to the manufacturer’s instructions. After transfer, membranes were blocked with 5% milk (Marvel) in TBS‐T (Tris‐buffered saline 10 mm, containing 0.5% Tween (v/v)) for 1 h at room temperature in a rocking surface. The membranes were then incubated for 1 h at room temperature or overnight at 4 °C on a rocking platform with the primary antibody at the working concentration in 5% TBS‐T milk. After incubation, membranes were washed three times for 10‐min intervals with TBS‐T and incubated with the secondary antibody at working concentration in 5% TBS‐T milk for 1 h at room temperature in a rocking platform. The membranes were then washed two times at intervals of 10 min with TBS‐T and one time with TBS for 10 min and were ready for development. Membranes were developed with enhanced chemiluminescence (ECL), using Pierce ECL western blotting substrate (Thermo Fisher Scientific), according to the manufacturer’s instructions. Signal was detected using a Li‐COR C‐Digit Western blot Scanner and image studio Software (Lincoln, NE, USA).

Primary antibodies used were as follows: anti‐p16 (1 : 1000, 108349; Abcam) and anti‐β‐actin (1 : 10 000, A1978; Sigma‐Aldrich).

Secondary antibodies used were as follows: anti‐mouse IgG HRP‐conjugated (1 : 5000, GTX221667‐01; GeneTex, Irvine, CA, USA) and anti‐rabbit IgG HRP‐conjugated (1 : 3000, 7074S; Cell Signalling, London, UK).

### Statistical analysis

Statistical analysis was done using graphpad prism 8 Software (San Diego, CA, USA). Comparison of two groups was done using the unpaired *t*‐test. When the comparison included more than two groups, one‐way ANOVA (analysis of variance) was performed. A *P* value < 0.05 was considered as statistically significant. The number of biological repeats is expressed as '*N*=' and the number of technical repeats as '*n*='.

## Conflict of interest

The authors declare no conflict of interest.

## Author contributions

SN, KDH and DWL conceived and designed the project. SN and DB performed the laboratory work and analysed the results. All authors were involved in writing and approving the manuscript.

## Data Availability

The data will be available from the corresponding author upon request.

## References

[1] Sharpless NE and Sherr CJ (2015) Forging a signature of *in vivo* senescence. Nat Rev Cancer 15, 397–408. 26105537 10.1038/nrc3960

[2] Tchkonia T , Zhu Y , van Deursen J , Campisi J and Kirkland JL (2013) Cellular senescence and the senescent secretory phenotype: therapeutic opportunities. J Clin Invest 123, 966–972. 23454759 10.1172/JCI64098 PMC3582125

[3] Coppe JP , Rodier F , Patil CK , Freund A , Desprez PY and Campisi J (2011) Tumor suppressor and aging biomarker p16(INK4a) induces cellular senescence without the associated inflammatory secretory phenotype. J Biol Chem 286, 36396–36403. 21880712 10.1074/jbc.M111.257071 PMC3196093

[4] Di Mitri D and Alimonti A (2016) Non‐cell‐autonomous regulation of cellular senescence in cancer. Trends Cell Biol 26, 215–226. 26564316 10.1016/j.tcb.2015.10.005

[5] Campisi J and d'Adda di Fagagna F (2007) Cellular senescence: when bad things happen to good cells. Nat Rev Mol Cell Biol 8, 729–740. 17667954 10.1038/nrm2233

[6] Malaquin N , Vercamer C , Bouali F , Martien S , Deruy E , Wernert N , Chwastyniak M , Pinet F , Abbadie C and Pourtier A (2013) Senescent fibroblasts enhance early skin carcinogenic events via a paracrine MMP‐PAR‐1 axis. PLoS One 8, e63607. 23675494 10.1371/journal.pone.0063607 PMC3651095

[7] Coppe JP , Patil CK , Rodier F , Sun Y , Munoz DP , Goldstein J , Nelson PS , Desprez PY and Campisi J (2008) Senescence‐associated secretory phenotypes reveal cell‐nonautonomous functions of oncogenic RAS and the p53 tumor suppressor. PLoS Biol 6, 2853–2868. 19053174 10.1371/journal.pbio.0060301 PMC2592359

[8] Casella G , Munk R , Kim KM , Piao Y , De S , Abdelmohsen K and Gorospe M (2019) Transcriptome signature of cellular senescence. Nucleic Acids Res 47, 7294–7305. 31251810 10.1093/nar/gkz555 PMC6698740

[9] Davalos AR , Coppe JP , Campisi J and Desprez PY (2010) Senescent cells as a source of inflammatory factors for tumor progression. Cancer Metastasis Rev 29, 273–283. 20390322 10.1007/s10555-010-9220-9 PMC2865636

[10] Hernandez‐Segura A , de Jong TV , Melov S , Guryev V , Campisi J and Demaria M (2017) Unmasking transcriptional heterogeneity in senescent cells. Curr Biol 27, 2652–2660.e4. 28844647 10.1016/j.cub.2017.07.033 PMC5788810

[11] Ozcan S , Alessio N , Acar MB , Mert E , Omerli F , Peluso G and Galderisi U (2016) Unbiased analysis of senescence associated secretory phenotype (SASP) to identify common components following different genotoxic stresses. Aging 8, 1316–1329. 27288264 10.18632/aging.100971 PMC4993333

[12] Lasry A and Ben‐Neriah Y (2015) Senescence‐associated inflammatory responses: aging and cancer perspectives. Trends Immunol 36, 217–228. 25801910 10.1016/j.it.2015.02.009

[13] Pernicova I and Korbonits M (2014) Metformin–mode of action and clinical implications for diabetes and cancer. Nat Rev Endocrinol 10, 143–156. 24393785 10.1038/nrendo.2013.256

[14] Fafian‐Labora J , Carpintero‐Fernandez P , Jordan SJD , Shikh‐Bahaei T , Abdullah SM , Mahenthiran M , Rodriguez‐Navarro JA , Niklison‐Chirou MV and O'Loghlen A (2019) FASN activity is important for the initial stages of the induction of senescence. Cell Death Dis 10, 318. 30962418 10.1038/s41419-019-1550-0 PMC6453932

[15] Freund A , Patil CK and Campisi J (2011) p38MAPK is a novel DNA damage response‐independent regulator of the senescence‐associated secretory phenotype. EMBO J 30, 1536–1548. 21399611 10.1038/emboj.2011.69 PMC3102277

[16] Herranz N , Gallage S , Mellone M , Wuestefeld T , Klotz S , Hanley CJ , Raguz S , Acosta JC , Innes AJ , Banito A *et al* (2015) mTOR regulates MAPKAPK2 translation to control the senescence‐associated secretory phenotype. Nat Cell Biol 17, 1205–1217. 26280535 10.1038/ncb3225 PMC4589897

[17] Watanabe S , Kawamoto S , Ohtani N and Hara E (2017) Impact of senescence‐associated secretory phenotype and its potential as a therapeutic target for senescence‐associated diseases. Cancer Sci 108, 563–569. 28165648 10.1111/cas.13184 PMC5406532

[18] Ishizaki T , Maekawa M , Fujisawa K , Okawa K , Iwamatsu A , Fujita A , Watanabe N , Saito Y , Kakizuka A , Morii N *et al* (1996) The small GTP‐binding protein Rho binds to and activates a 160 kDa Ser/Thr protein kinase homologous to myotonic dystrophy kinase. EMBO J 15, 1885–1893. 8617235 PMC450107

[19] Leung T , Chen XQ , Manser E and Lim L (1996) The p160 RhoA‐binding kinase ROK alpha is a member of a kinase family and is involved in the reorganization of the cytoskeleton. Mol Cell Biol 16, 5313–5327. 8816443 10.1128/mcb.16.10.5313 PMC231530

[20] Liao JK , Seto M and Noma K (2007) Rho kinase (ROCK) inhibitors. J Cardiovasc Pharmacol 50, 17–24. 17666911 10.1097/FJC.0b013e318070d1bd PMC2692906

[21] Schaafsma D , Gosens R , Zaagsma J , Halayko AJ and Meurs H (2008) Rho kinase inhibitors: a novel therapeutical intervention in asthma? Eur J Pharmacol 585, 398–406. 18410919 10.1016/j.ejphar.2008.01.056

[22] Honjo M and Tanihara H (2018) Impact of the clinical use of ROCK inhibitor on the pathogenesis and treatment of glaucoma. Jpn J Ophthalmol 62, 109–126. 29445943 10.1007/s10384-018-0566-9

[23] Wang Y , Lu Y , Chai J , Sun M , Hu X , He W , Ge M and Xie C (2017) Y‐27632, a Rho‐associated protein kinase inhibitor, inhibits systemic lupus erythematosus. Biomed Pharmacother 88, 359–366. 28122300 10.1016/j.biopha.2017.01.069

[24] Masumoto A , Hirooka Y , Shimokawa H , Hironaga K , Setoguchi S and Takeshita A (2001) Possible involvement of Rho‐kinase in the pathogenesis of hypertension in humans. Hypertension 38, 1307–1310. 11751708 10.1161/hy1201.096541

[25] Shibuya M , Hirai S , Seto M , Satoh S and Ohtomo E (2005) Effects of fasudil in acute ischemic stroke: results of a prospective placebo‐controlled double‐blind trial. J Neurol Sci 238, 31–39. 16005902 10.1016/j.jns.2005.06.003

[26] Nakata J , Akiba Y , Nihara J , Thant L , Eguchi K , Kato H , Izumi K , Ohkura M , Otake M , Kakihara Y *et al* (2020) ROCK inhibitors enhance bone healing by promoting osteoclastic and osteoblastic differentiation. Biochem Biophys Res Commun 526, 547–552. 32192772 10.1016/j.bbrc.2020.03.033

[27] Clayton NS and Ridley AJ (2020) Targeting Rho GTPase signaling networks in cancer. Front Cell Dev Biol 8, 222. 32309283 10.3389/fcell.2020.00222 PMC7145979

[28] Lau L , Porciuncula A , Yu A , Iwakura Y and David G (2019) Uncoupling the senescence‐associated secretory phenotype from cell cycle exit via interleukin‐1 inactivation unveils its protumorigenic role. Mol Cell Biol 39, e00586‐18. 30988157 10.1128/MCB.00586-18 PMC6549465

[29] McCarthy DA , Clark RR , Bartling TR , Trebak M and Melendez JA (2013) Redox control of the senescence regulator interleukin‐1alpha and the secretory phenotype. J Biol Chem 288, 32149–32159. 24062309 10.1074/jbc.M113.493841 PMC3820855

[30] Banerjee S and McGee DW (2016) ROCK activity affects IL‐1‐induced signaling possibly through MKK4 and p38 MAPK in Caco‐2 cells. In Vitro Cell Dev Biol Anim 52, 878–884. 27173611 10.1007/s11626-016-0050-0

[31] Chapman S , Liu X , Meyers C , Schlegel R and McBride AA (2010) Human keratinocytes are efficiently immortalized by a Rho kinase inhibitor. J Clin Invest 120, 2619–2626. 20516646 10.1172/JCI42297 PMC2898606

[32] Wei L , Surma M , Shi S , Lambert‐Cheatham N and Shi J (2016) Novel insights into the roles of Rho kinase in cancer. Arch Immunol Ther Exp 64, 259–278. 10.1007/s00005-015-0382-6 PMC4930737 26725045

[33] Chapman S , McDermott DH , Shen K , Jang MK and McBride AA (2014) The effect of Rho kinase inhibition on long‐term keratinocyte proliferation is rapid and conditional. Stem Cell Res Ther 5, 60. 24774536 10.1186/scrt449 PMC4055106

[34] Campisi J (2013) Aging, cellular senescence, and cancer. Annu Rev Physiol 75, 685–705. 23140366 10.1146/annurev-physiol-030212-183653 PMC4166529

[35] Demaria M , Ohtani N , Youssef SA , Rodier F , Toussaint W , Mitchell JR , Laberge RM , Vijg J , Van Steeg H , Dollé ME *et al* (2014) An essential role for senescent cells in optimal wound healing through secretion of PDGF‐AA. Dev Cell 31, 722–733. 25499914 10.1016/j.devcel.2014.11.012 PMC4349629

[36] Soto‐Gamez A and Demaria M (2017) Therapeutic interventions for aging: the case of cellular senescence. Drug Discov Today 22, 786–795. 28111332 10.1016/j.drudis.2017.01.004

[37] Guo Y , Ayers JL , Carter KT , Wang T , Maden SK , Edmond D , Newcomb PP , Li C , Ulrich C , Yu M *et al* (2019) Senescence‐associated tissue microenvironment promotes colon cancer formation through the secretory factor GDF15. Aging Cell 18, e13013. 31389184 10.1111/acel.13013 PMC6826139

[38] Ortiz‐Montero P , Londono‐Vallejo A and Vernot JP (2017) Senescence‐associated IL‐6 and IL‐8 cytokines induce a self‐ and cross‐reinforced senescence/inflammatory milieu strengthening tumorigenic capabilities in the MCF‐7 breast cancer cell line. Cell Commun Signal 15, 17. 28472950 10.1186/s12964-017-0172-3 PMC5418812

[39] Cahu J , Bustany S and Sola B (2012) Senescence‐associated secretory phenotype favors the emergence of cancer stem‐like cells. Cell Death Dis 3, e446. 23254289 10.1038/cddis.2012.183 PMC3542619

[40] Acosta JC , Banito A , Wuestefeld T , Georgilis A , Janich P , Morton JP , Athineos D , Kang TW , Lasitschka F , Andrulis M *et al* (2013) A complex secretory program orchestrated by the inflammasome controls paracrine senescence. Nat Cell Biol 15, 978–990. 23770676 10.1038/ncb2784 PMC3732483

[41] Gardner SE , Humphry M , Bennett MR and Clarke MC (2015) Senescent vascular smooth muscle cells drive inflammation through an interleukin‐1alpha‐dependent senescence‐associated secretory phenotype. Arterioscler Thromb Vasc Biol 35, 1963–1974. 26139463 10.1161/ATVBAHA.115.305896 PMC4548545

[42] Orjalo AV , Bhaumik D , Gengler BK , Scott GK and Campisi J (2009) Cell surface‐bound IL‐1alpha is an upstream regulator of the senescence‐associated IL‐6/IL‐8 cytokine network. Proc Natl Acad Sci USA 106, 17031–17036. 19805069 10.1073/pnas.0905299106 PMC2761322

[43] Georgilis A , Klotz S , Hanley CJ , Herranz N , Weirich B , Morancho B , Leote AC , D'Artista L , Gallage S , Seehawer M *et al* (2018) PTBP1‐mediated alternative splicing regulates the inflammatory secretome and the pro‐tumorigenic effects of senescent cells. Cancer Cell 34, 85–102.e9. 29990503 10.1016/j.ccell.2018.06.007 PMC6048363

[44] Kang W , Shang L , Wang T , Liu H and Ge S (2018) Rho‐kinase inhibitor Y‐27632 downregulates LPS‐induced IL‐6 and IL‐8 production via blocking p38 MAPK and NF‐kappaB pathways in human gingival fibroblasts. J Periodontol 89, 883–893. 29630729 10.1002/JPER.17-0571

[45] Liu X , Ory V , Chapman S , Yuan H , Albanese C , Kallakury B , Timofeeva OA , Nealon C , Dakic A , Simic V *et al* (2012) ROCK inhibitor and feeder cells induce the conditional reprogramming of epithelial cells. Am J Pathol 180, 599–607. 22189618 10.1016/j.ajpath.2011.10.036 PMC3349876

[46] Faget DV , Ren Q and Stewart SA (2019) Unmasking senescence: context‐dependent effects of SASP in cancer. Nat Rev Cancer 19, 439–453. 31235879 10.1038/s41568-019-0156-2

[47] McGregor F , Muntoni A , Fleming J , Brown J , Felix DH , MacDonald DG , Parkinson EK and Harrison PR (2002) Molecular changes associated with oral dysplasia progression and acquisition of immortality: potential for its reversal by 5‐azacytidine. Cancer Res 62, 4757–4766. 12183435

[48] Rheinwald JG and Green H (1975) Serial cultivation of strains of human epidermal keratinocytes: the formation of keratinizing colonies from single cells. Cell 6, 331–343. 1052771 10.1016/s0092-8674(75)80001-8

[49] Grayson AK , Hearnden V , Bolt R , Jebreel A , Colley HE and Murdoch C (2018) Use of a Rho kinase inhibitor to increase human tonsil keratinocyte longevity for three‐dimensional, tissue engineered tonsil epithelium equivalents. J Tissue Eng Regen Med 12, e1636–e1646. 29048773 10.1002/term.2590

